# Selection and evaluation of housekeeping genes as endogenous controls for quantification of mRNA transcripts in *Theileria parva* using quantitative real-time polymerase chain reaction (qPCR)

**DOI:** 10.1371/journal.pone.0196715

**Published:** 2018-05-04

**Authors:** Teboho N. Tsotetsi, Nicola E. Collins, Marinda C. Oosthuizen, Kgomotso P. Sibeko-Matjila

**Affiliations:** Department of Veterinary Tropical Diseases, Vectors and Vector-borne Diseases Research Programme, Faculty of Veterinary Science, University of Pretoria, Onderstepoort, Gauteng Province, South Africa; Institut national de la santé et de la recherche médicale - Institut Cochin, FRANCE

## Abstract

The reliability of any quantitative real-time polymerase chain reaction (qPCR) experiment can be seriously compromised by variations between samples as well as between PCR runs. This usually result from errors in sample quantification, especially with samples that are obtained from different individuals and tissues and have been collected at various time intervals. Errors also arise from differences in qPCR efficiency between assays performed simultaneously to target multiple genes on the same plate. Consequently, the derived quantitative data for the target genes become distorted. To avoid this grievous error, an endogenous control, with relatively constant transcription levels in the target individual or tissue, is included in the qPCR assay to normalize target gene expression levels in the analysis. Several housekeeping genes (HKGs) have been used as endogenous controls in quantification studies of mRNA transcripts; however, there is no record in the literature of the evaluation of these genes for the tick-borne protozoan parasite, *Theileria parva*. Importantly, the expression of these genes should be invariable between different *T*. *parva* stocks, ideally under different experimental conditions, to gain extensive application in gene expression studies of this parasite. Thus, the expression of several widely used HKGs was evaluated in this study, including the genes encoding β**-**actin, glyceraldehyde-3-phosphate dehydrogenase (GAPDH), 28S rRNA, cytochrome b and fructose-2.6-biphosphate aldolase (F6P) proteins. The qPCR analysis revealed that the expression of genes encoding cytochrome b, F6P and GAPDH varied considerably between the two *T*. *parva* stocks investigated, the cattle-derived *T*. *parva* Muguga and the buffalo-derived *T*. *parva* 7014. 28S rRNA and β**-**actin gene expression was the most stable; thus, these genes were considered suitable candidates to be used as endogenous control genes for mRNA quantification studies in *T*. *parva*.

## Introduction

A number of techniques are used to quantify a given mRNA transcript, including RNase protection assays, northern blotting, reserve transcription quantitative real-time PCR (RT-qPCR), and *in situ* hybridization [1–4]. However, the quantitative real-time PCR (qPCR) based methods have added advantages compared to its counterparts as they have the ability to quantify low target copy numbers from small quantities of RNA, and to identify minor changes in mRNA expression levels in these samples [5]. Thus, this method has become a common technique for measuring mRNA levels, especially of low copy number transcripts, particularly in the absence of suitable alternative assays [5]. In addition to the remarkable sensitivity, the benefits of qPCR over other quantitative methods extends to: large dynamic range, the potential for high throughput, reduced probabilities of variability, complete automated process, as well as elimination of post-reaction analyses, which considerably reduces contamination and assay time [6]. Hence, the technology has established itself as the gold standard for medium throughput gene expression analysis [7] and it has become the method of choice for validating data generated from RNA sequencing (RNA-seq) [8,9].

Since RNA is not a suitable template for qPCR because of its volatile nature, quantification of mRNA requires cDNA synthesis from the RNA template, through an *in vitro* reverse transcription process. The reverse transcription can be performed separate or coupled with qPCR in a RT-qPCR reaction. Unfortunately, in both processes (qPCR and RT-qPCR), quantification of mRNA transcripts is subject to errors when the amount of starting material varies between samples investigated, which may arise from poor RNA integrity, differences in efficiency of reverse transcription and cDNA sample loading [10–13]. Such errors are exacerbated when samples are obtained at different time intervals, from various individuals and/or tissues samples. Most crucial is that the resultant expression profiles for the target genes are flawed [14,15]. However, these errors can be normalized by including an endogenous control (reference gene), with constitutive stable expression between investigated individuals or tissues, in the qPCR assay [16]. Besides variations in sample quantities and quality, there are also experimental errors resulting from multi-step process involved in sample preparation, which require normalization in order to obtain reliable results [17]. Thus, there is a need to control for variations arising from RNA extraction and processing. The common normalization approach for RNA preparation processes is to measure an internal control, usually a housekeeping gene [12]. Although this is important, usually the normalization of RNA preparation processes is overlooked.

Endogenous control genes are usually selected from housekeeping or maintenance genes because of their invariable expression levels. Housekeeping genes (HKGs) are genes that occur in all cells since they encode proteins that are responsible for the basic function of the cell [18]. Their expression is usually constant in all cells of an organism and is expected to remain the same under various intracellular conditions. The most common genes used as endogenous controls in RNA quantitative studies include genes encoding α- actin, β- actin, 18S rRNA, 28S rRNA, albumin, cyclophilins, glyceraldehyde-3-phosphate dehydrogenase (GAPDH) and tubulins proteins [18].

Since qPCR is only suitable for medium throughput studies, RNA-seq provides robust high throughput RNA transcript quantification and transcriptomics information for identification of differentially expressed transcripts. Similar to qPCR, RNA-seq experiments can be influenced by variability resulting from multi-process technical procedures such as RNA extraction, cDNA synthesis and modification for generation of fragments for libraries construction [19,20]. It is thus essential for transcriptome profiles generated from RNA-seq data to be validated. These transcriptome profiles are usually validated employing techniques such as qPCR, DNA microarray and in some instances the quantitative proteomic approach; although, qPCR is usually the preferred and popular method.

Housekeeping genes have been used widely as endogenous control genes expression studies; however, evaluation of the expression profiles of these genes has not been reported in *T*. *parva*. *Theileria parva* is a haemoprotozoan parasite occurring in 11 countries in east, central and southern Africa [21]. It is principally transmitted by the brown ear ticks *Rhipicephalus appendiculatus* and *R*. *zambeziensis* and its natural reservoir host is the African buffalo (*Syncerus caffer*) [22]. *Theileria parva* infections in cattle are associated with three lymphoproliferative disease syndromes; East Coast fever (ECF) and January disease caused by cattle-derived *T*. *parva*, and Corridor disease resulting from infection with buffalo-derived *T*. *parva* [23]. Due to high mortalities (over 80% in susceptible animals), *T*. *parva* infections, particularly ECF, are the major animal health and economic constraint undermining efforts to improve the productivity of cattle in affected countries in Africa [24]. The disease especially affect the more productive and most susceptible European and improved zebu breeds, killing over 1 million cattle annually; hence it is reportedly the single biggest killer of cattle in affected countries [24,25].

The effective control of cattle theleriosis caused by *T*. *parva* infections requires a better understanding of the parasite biology and pathogenicity, of which both may be influenced by gene expression in different hosts and parasite developmental stages. To facilitate gene expression work, it is important to identify reliable endogenous control genes to avoid errors already mentioned above. Thus in this study, some of the widely used HKGs were evaluated to determine their fitness as candidate endogenous controls for *T*. *parva* gene expression studies. The *T*. *parva* HKGs evaluated in this study encode β**-**actin, GAPDH, 28S rRNA, cytochrome b and fructose-2.6-biphosphate aldolase (F6P) proteins.

## Materials and methods

### Source of RNA, RNA isolation and cDNA synthesis

Bovine and buffalo lymphoblastoid cell cultures infected with the schizont developmental stage of *T*. *parva* stocks Muguga and 7014, maintained at the Institute of Tropical Medicine in Antwerp, Belgium, were made available to the study by Dr Dirk Geysen (Department of Biomedical Sciences, Institute of Tropical Medicine, Belgium) and were used as the source of RNA. Total RNA was isolated from *T*. *parva-*infected cell cultures using the RNeasy plus mini kit (Qiagen, Germany), according to the manufacturer’s instructions. A maximum of 1x10^7^ cells was used for a single extraction. The extracted RNA was eluted in 50 μl of RNase-free water provided in the kit.

The quality of the RNA was determined using a NanoDrop ND-1000 spectrophotometer (NanoDrop Technologies). To minimize adverse effects of protein contamination, only the RNA samples with 260/280 ratio between 1.9 and 2.1 and 260/230 ratio greater than 2.0 were used for the downstream analyses. The cDNA was synthesized from total RNA using the iScript Advanced cDNA Synthesis kit (Bio-Rad, USA) according to the manufacturer’s instructions.

### Quantification of *T*. *parva* cDNA by qPCR using a standard curve

The total RNA extracted from *T*. *parva*-infected cell cultures is usually contaminated with host RNA and at the time of study there was no assay available to determine the copy number or concentration of the parasite RNA material using cDNA prepared from *T*. *parva*-infected cell cultures. Thus, pure blue tongue virus (BTV) cDNA of known concentration, provided by Dr Peter Coetzee (Department of Veterinary Tropical Diseases, University of Pretoria, South Africa), was used to generate a standard curve by qPCR; this standard curve was then used to determine the concentration of *T*. *parva* cDNA by comparison with qPCR amplification of *T*. *parva* 28S rRNA. Prior to quantification, the efficiencies of the two qPCR assays were evaluated using cDNA prepared from *T*. *parva*-infected cell cultures and BTV cDNA. The BTV target gene, encoding the variable outer shell protein (VP2), was amplified using the following forward and reverse primers: F 5’ CGG ACC GCA TTA TGG TAT AAC C 3’ and R 5’ ACT CTT GTG TCT CGT ACT TTC AAC 3’ [26]. The BTV cDNA concentrations for the standard curve dilutions ranged from 1000 ng/μl to 0.1 ng/μl. The *T*. *parva* 28S rRNA gene target was amplified using the primers shown in Table 1.

**Table 1:** Primer sequences for qPCR amplification of five candidate *T*. *parva* reference genes, the membrane transporter gene used for evaluation of qPCR assay precision and the four differentially expressed genes.

Accession No.	Gene description	Primer sequence 5’-3’	Amplicon length (bp)	Primer melting temperature (°C)
**Housekeeping genes:**				
**XM_760535.1**	Fructose-2.6-bisphosphate	Forward- TATGCGCTGGTGTGTCAGTC Reverse- CACTCCTCTGCTGAATGGCT	86	58
**AB499089.1**	Cytochrome b	Forward- GGTAAGACCCTGTGCACCTT Reverse- CACCTCCATGTCGTCTCACC	84	58
**L28998.1**	28S rRNA	Forward- CGACTGTCCGTGAAAAGGGA Reverse- AACCTTGGAGACCTGATGCG	80	58
**XM_760378.1**	β-Actin	Forward- ATGTTGCAATCCAGGCCGTA Reverse- GTGGGTGACACCATCTCCAG	86	58
**XM_760333.1**	Glyceraldehyde-3-phosphate dehydrogenase	Forward- CCCCTCAATGACGCCAGAAT Reverse- TTCCTCGAGCAGCAATGTGT	88	58
**The gene used for evaluation of qPCR precision:**					
**XM_758301.1**	Membrane transporter	Forward- TGACTGCACACCACTTCTTC Reverse- CAGTTAAACCTGACACCGCT	120	58
**Genes differentially expressed in *T*. *parva* Muguga and *T*. *parva* 7014:**				
**XM_757780.1**	Hypothetical protein	Forward- GTGAGAAGGGAGTCAGATTCG Reverse- CTTGATTGACTCAAATACATGCGA	80	58
**XM_758397.1**	Apical membrane antigen-1	Forward- TAAATGACAGCGCTCAGGAC Reverse- GACGAGTAGTACGTGAAGCC	88	58
**XM_757777.1**	DNA-directed polymerase iii subunit rpc2	Forward- ACCTGGAAATCAGCCCAATG Reverse- ATGTGTTTCTTGGGCTCTGG	87	58

All qPCR experiments were performed employing the ABI StepOnePlus™ system and software (Applied Biosystems, USA). The qPCR reaction mixture contained 2.5 μl of template cDNA (~36.0 ng/μl), 10 μl of 2× Platinum SYBR Green SuperMix-UDG (Life technologies, USA), and 0.2μM stock concentration of each gene-specific primer in a final volume of 20 μl. All qPCR reactions were performed under the following conditions: UDG activation for 2 minutes at 50°C, denaturation for 2 minutes at 95°C, and 40 amplification cycles of denaturation for 10 seconds at 95°C, primer annealing for 10 seconds at 58°C and final extension for 1 minute at 72°C. The specificity of the qPCR reaction for each amplified product was verified by melting curve analysis, which was carried out as follows: 15 seconds at 95°C, 1 minute at 60°C, (with 20°C/s transition rate), and then ramping to 95°C (at 0.2°C/s transition rate) and 15 seconds at 95°C. For the BTV gene standard curve, triplicate samples of each dilution were subjected to qPCR, as required by MIQE (Minimal information for Publication of Quantitative Real-time PCR Experiments) guidelines [27]. The *T*. *parva* 28S rRNA gene target region was also amplified from three replicates. In each qPCR run, a ‘no template’ control as well as an uninfected bovine cDNA control were included.

### Primer design and quantification of expression of *T*. *parva* housekeeping genes

For identification of reliable endogenous/reference genes for normalization of *T*. *parva* gene expression studies by qPCR, five widely used HKGs were selected, including genes that code for 28S rRNA, β**-**actin, cytochrome b, fructose-2.6-biphosphate aldolase (F6P) and glyceraldehyde-3-phosphate dehydrogenase (GAPDH) proteins. Primer-Blast software (NCBI, USA) was employed to design oligonucleotide primers for amplification of the selected HKGs using default parameters. Wherever possible, the primers were designed spanning an intron to allow detection of any genomic DNA contamination based on PCR product size variation. To select primers for specific amplification of *T*. *parva* HKGs, the retrieved primer pairs were analysed using BLAST (Basic sequence alignment tool: https://blast.ncbi.nlm.nih.gov/Blast.cgi) to search for matches with other HKGs. The primers were synthesized by Life Technologies (USA) and the primer sequences are presented in Table 1.

For quantification of HKGs in cDNA from *T*. *parva* Muguga and *T*. *parva* 7014 infected cell cultures, qPCRs were performed as described above using the primers shown in Table 1. The qPCR amplification of the target region of each HKG was performed from two biological replicates and three technical replicates were analyzed for each biological replicate in two independent runs. The specificity of the qPCR for each amplified product was verified by melting curve analysis and, in addition, the qPCR amplicon size was confirmed by agarose gel electrophoresis.

### Evaluation of expression stability of housekeeping genes

The expression stability of the five HKGs under investigation in the two *T*. *parva* isolates was determined using the cycle of quantification (Cq) values obtained from qPCR. The qPCR data was analyzed employing RefFinder (http://leonxie.esy.es/RefFinder/). The most stably expressed candidate genes within and between the test groups are those with the lowest variation values.

### Evaluation of qPCR assays for precision and reproducibility

The precision of the qPCR assay was determined using a randomly selected gene, that codes for a *T*. *parva* transmembrane protein (accession number XM_758301.1). Amplification was performed across a 10-fold dilution series (36.0 ng/μl to 0.0036 ng/μl) prepared from *T*. *parva* Muguga and 7014 cDNA samples, using the PCR primers shown in Table 1. The Cq values for each isolate were separately plotted against the cDNA concentrations. The linearity of amplification for the selected HKGs was determined by the *R*^*2*^ values of each dilution series, and accepted at the efficiency range of between 90 and 110% (results not shown).

Intra-assay (across the plate) and inter-assay (between plates) variability was determined according to MIQE guidelines [27]. The mean of Cq values, standard deviation (SD) and coefficient of variation (CV) of Cq values were calculated separately for amplification of representative target genes from *T*. *parva* Muguga and *T*. *parva* 7014 cDNA. The intra- and inter-assay variability were assessed using the CV value, which was determined through dividing the SD by the mean Cq value; the resulting value was multiplied by 100 to express CV as a percentage. In order to determine the precision of the resulting data, intra and inter assay variation analyses were performed for expression of HKGs between *T*. *parva* Muguga and 7014, using the Student’s *t*-test Paired Two Samples for Means analysis.

### Evaluation of the selected endogenous genes to normalize qPCR data of differentially expressed genes

The reliability of β-actin and 28S rRNA to normalize expression data for *T*. *parva* genes was evaluated using three genes differentially expressed in *T*. *parva* 7014 and *T*. *parva* Muguga, as previously identified by RNA-seq analysis (Table 1). For the gene expression quantification experiments *T*. *parva* Muguga was used as the reference sample while *T*. *parva* 7014 was used as the test sample. The selected genes were amplified from both *T*. *parva* Muguga and *T*. *parva* 7014 using qPCR conditions described above for quantification of expression of *T*. *parva* housekeeping genes. Each gene target and the two selected HKGs (28S rRNA and β-actin) were run in triplicate and the mean Cq values were used for analysis. Each target gene in each sample was normalized by subtracting the mean Ct value of the endogenous control gene (β-actin or 28S rRNA) from the mean Ct value of the target gene (C_tmean_ target gene -C_tmean_ endogenous control genes), thereafter the difference of each normalized target gene was obtained (ΔCt 7014 - ΔCt Muguga) and the fold difference was calculated using the equation 2^-ΔΔC^_T_. The up-regulated and down-regulated genes amplification data was analyzed with relative quantification min/max confidence of 95%.

## Results

### Real-time PCR amplification efficiency and quantification of *T*. *parva* cDNA

**Fig 1:**
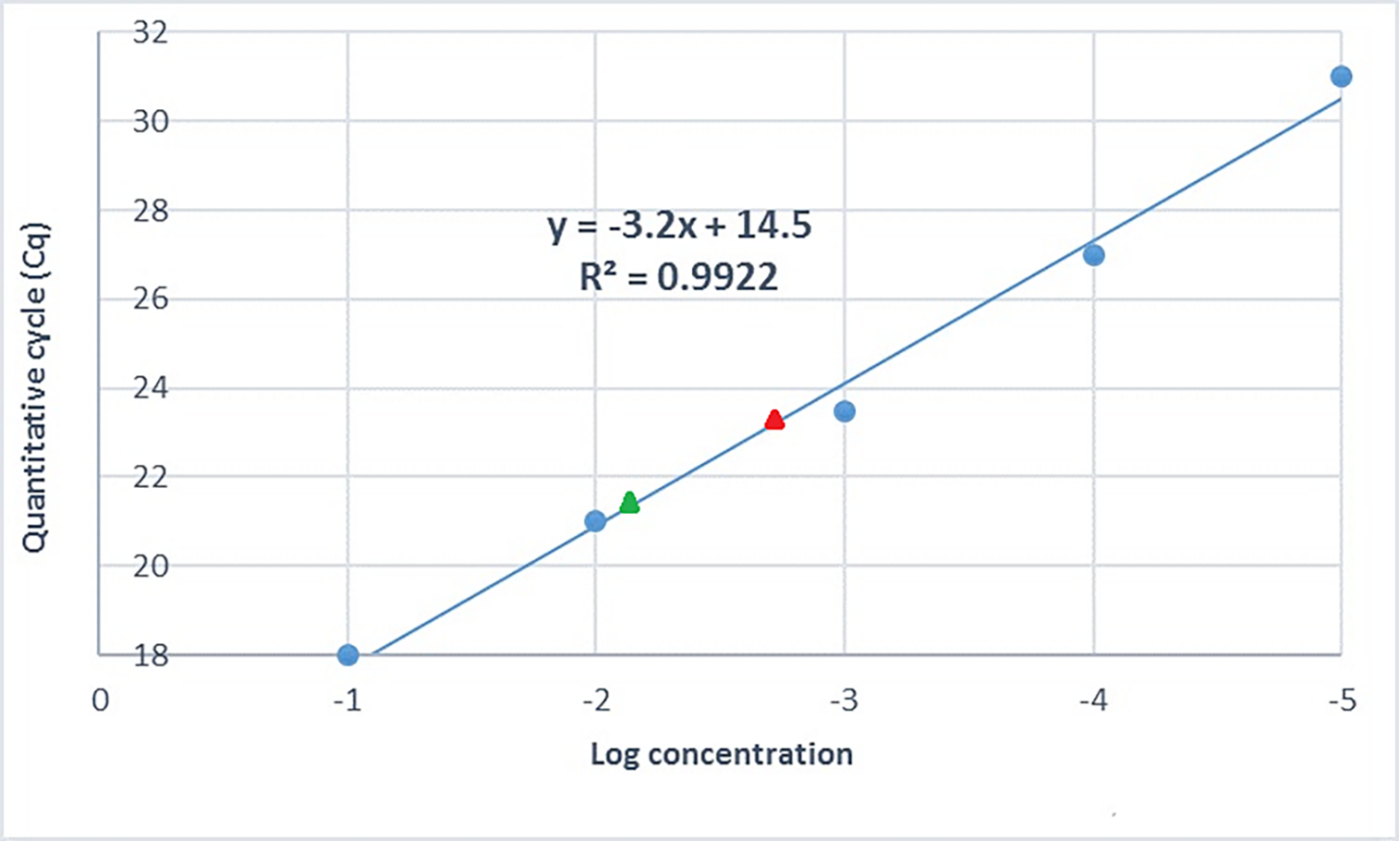
A standard curve generated by amplification of the VP2 gene from a 10-fold dilution series of BTV cDNA of known concentration (1000 to 0.1ng/μl), used to determine the concentration of *T*. *parva* cDNA by comparison with qPCR amplification of *T*. *parva* 28S rRNA from parasite isolates *T*. *parva* Muguga (designated with a red triangle) and *T*. *parva* 7014 (designated with a green triangle).

A standard curve generated from amplification of the BTV gene coding for the VP2 protein was used to determine the concentration of the *T*. *parva* cDNA prepared from total RNA extracted from cell cultures, by quantification of the *T*. *parva* 28S rRNA gene. Prior to quantifying the parasite cDNA, the working efficiency of the standard curve generated using the VP2 gene of BTV was evaluated and determined to be 97%, with a correlation coefficient (R^2^) value of 0.992 and a slope of -3.2 (Fig 1). Comparison of the BTV qPCR and *T*. *parva* 28S rRNA qPCR assays showed that the two had comparable amplification efficiencies (results not shown). The parasite cDNA concentration interpolated from the standard curve was 36.03 ng/μl for *T*. *parva* Muguga and 127.77 ng/μl for *T*. *parva* 7014. Subsequently, *T*. *parva* 7014 cDNA samples were diluted to adjust the concentration such that they were equivalent to the *T*. *parva* Muguga cDNA concentration in order to eliminate distorted output in downstream gene expression analysis.

### Confirmation of primer specificity for *T*. *parva* housekeeping genes

**Fig 2:**
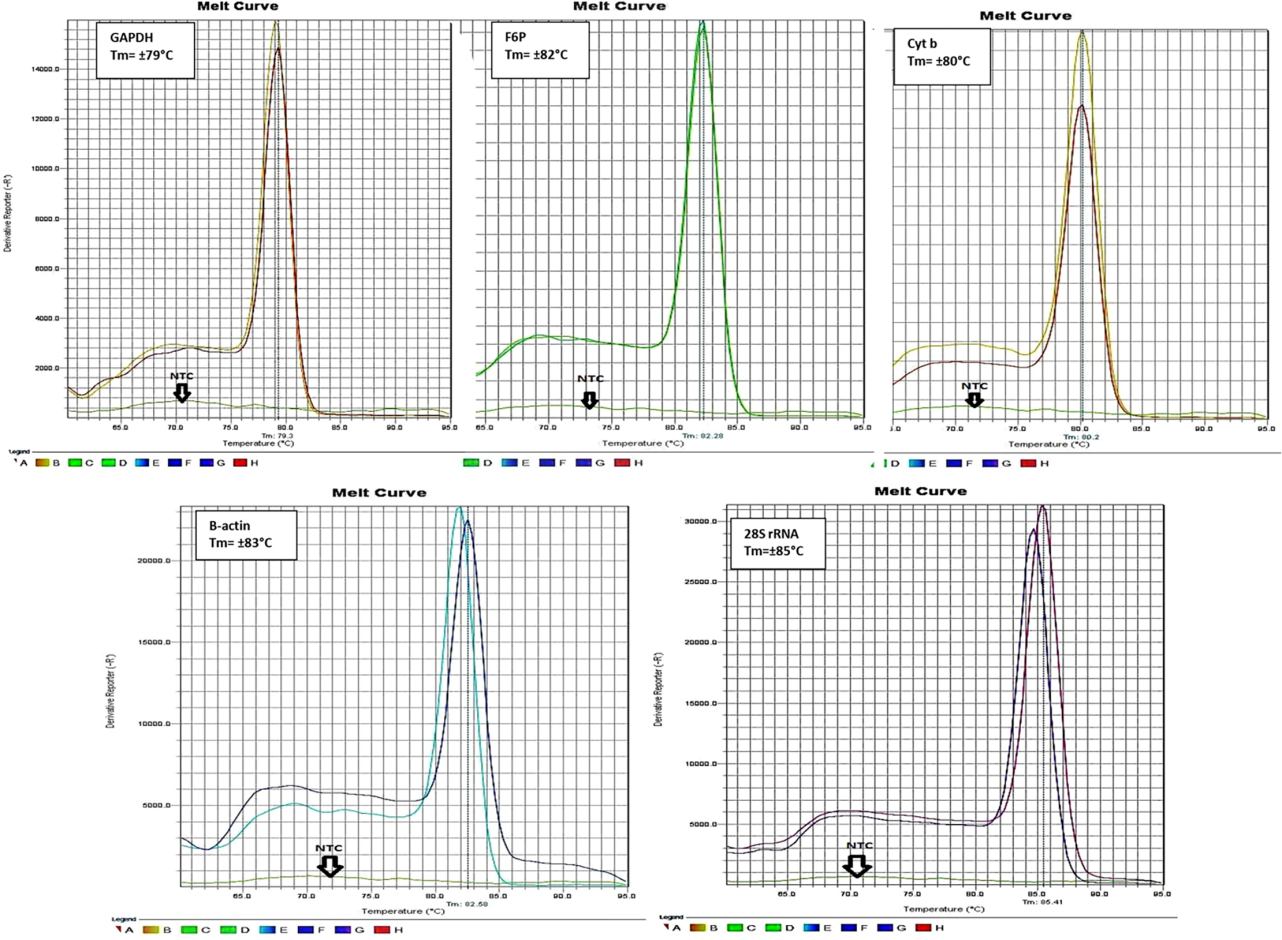
Specificity of real-time PCR amplification: Melting curves generated after amplification of five candidate *T*. *parva* reference genes showing a single melting peak for each product. Each experiment included two biological replicates of cDNA prepared from RNA isolated from cell cultures infected with *T*. *parva* Muguga and *T*. *parva* 7014 and a no template control (NTC).

The specificity of the primers designed for amplification of *T*. *parva* HKGs was determined by melting curve analysis. Single product-specific melting peaks were detected at different melting temperatures (Tm’s) for each gene product: F6P at 86°C, 28S rRNA at 85°C, β**-**actin at 81°C, cytochrome b at 80°C and GAPDH at 79°C (Fig 2). In addition, agarose gel electrophoresis of qPCR products revealed single amplicons of the expected length; the amplicon sizes were 88 bp for GAPDH, 86 bp for F6P, 86 bp for β-actin, 84 bp for cytochrome b and 80 bp for 28S rRNA (results not shown).

### Analysis of stability of expression of *T*. *parva* housekeeping genes

The web-based software program, RefFinder, was employed to analyze the expression stability of the HKGs and rank them accordingly (http://www.leonxie.com/referencegene.php). ReFfinder evaluates the reference gene stability through integrated computational programs, including geNorm, Normfinder, BestKeeper. The program then uses the comparative delta C_t_ method to compare and rank specific candidate endogenous control genes. Subsequently it then assigns an appropriate weight to each gene and calculates the geometric mean of the gene weights for the overall final ranking, according to the rankings from each program. Finally, each gene is assigned an expression stability value (*M*); genes with the high *M* value are considered to have unstable expression while genes with the lowest *M* value are the most stable and regarded suitable endogenous control gene candidates [28].

**Fig 3:**
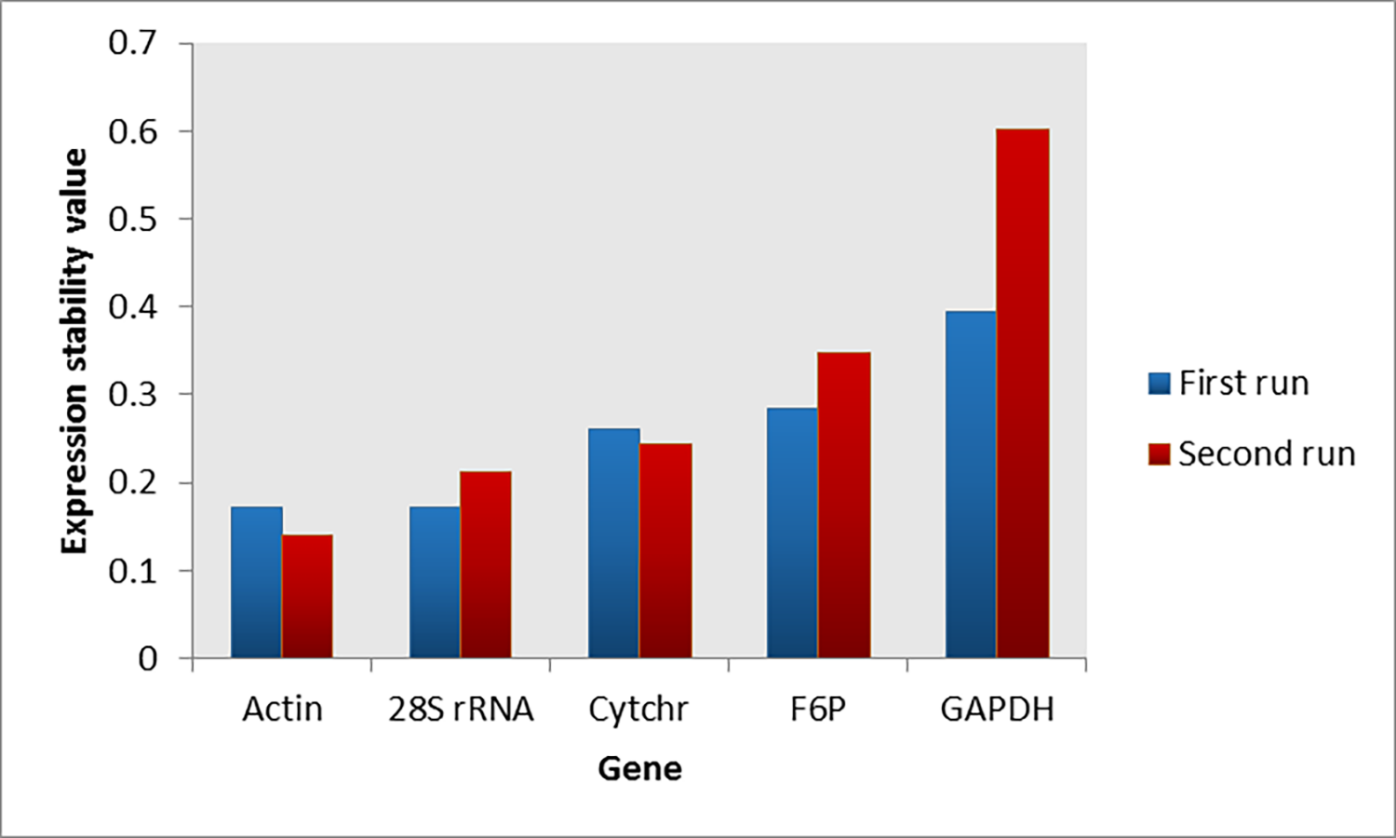
Expression stability rankings of five candidate *T*. *parva* reference genes obtained from two qPCR runs. Actin = β-actin; Cytchr = cytochrome b; F6P = fructose-2.6-biphosphate aldolase; GAPDH = glyceraldehyde phosphate dehydrogenase.

Two separate real-time PCR runs were performed for analysis of expression stability of the HKGs. In the first run, the expression stability values of 28S rRNA (*M* = 0.171) and β**-**actin (*M* = 0.171) were the lowest (Fig 3). Consistently, in the second run, the same two genes, 28S rRNA (*M* = 0.141) and β**-**actin (*M* = 0.213), had the lowest expression stability values (Fig 3). Table 2 illustrates the overall expression stability of the HKGs in both runs according to each computational program.

**Table 2:** Gene expression stability of five candidate *T*. *parva* reference genes as assessed by RefFinder.

Ranking Order (Better—Good—Average)					
Method	1	2	3	4	5
Delta C_T_	β-actin	Cytochrome b	28S rRNA	F6P	GAPDH
Bestkeeper	28S rRNA	β-actin	Cytochrome b	F6P	GAPDH
Normfinder	β-actin	Cytochrome b	28S rRNA	F6P	GAPDH
geNorm	Cytochrome b/28S rRNA	-	β-actin	F6P	GAPDH
**Recommended comprehensive ranking**	**β-actin**	**28S rRNA**	**Cytochrome b**	**F6P**	**GAPDH**

### Intra- and inter-assay variation analysis

**Fig 4:**
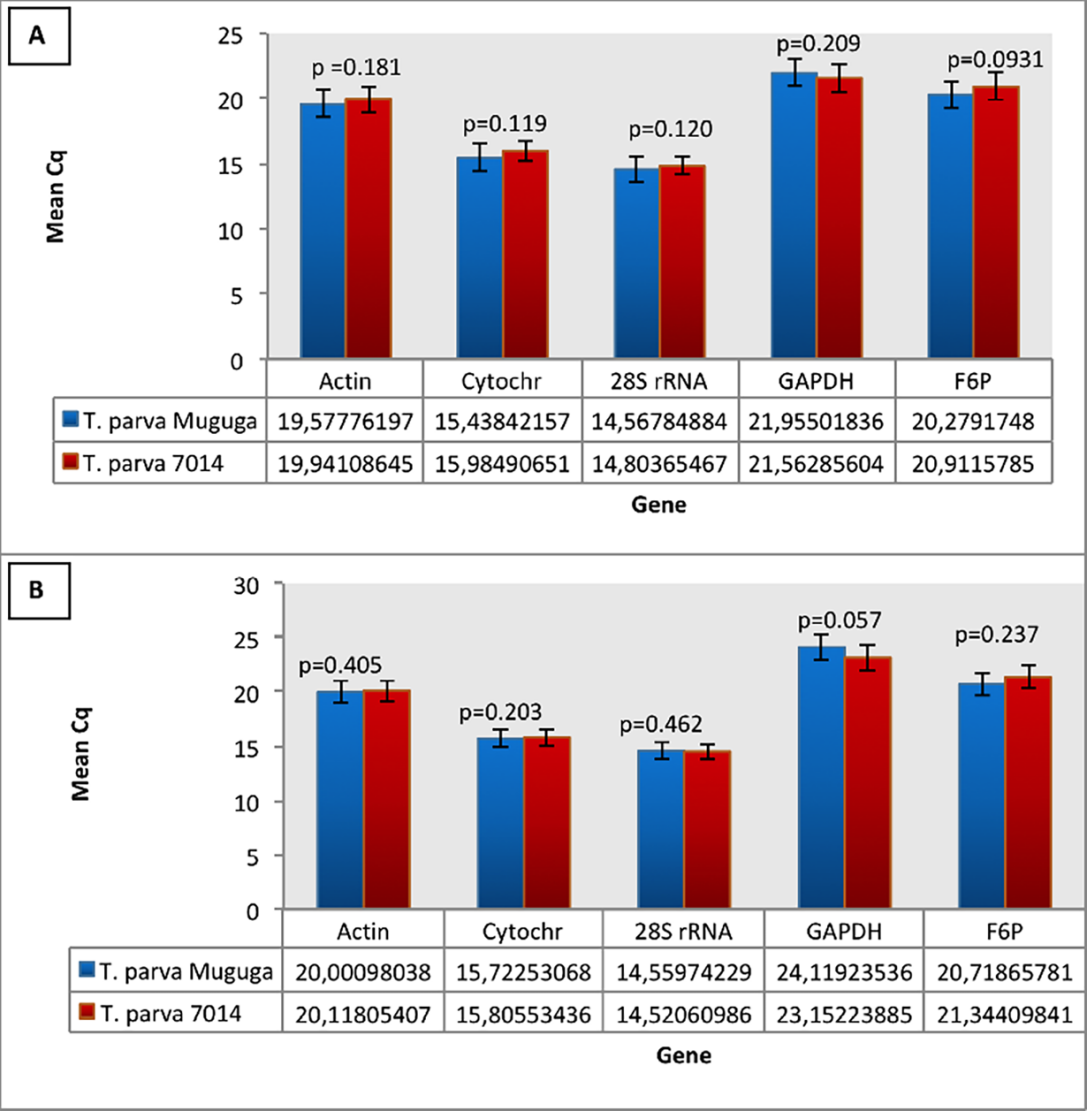
Student’s *t*-test results for analysis of intra-assay variations in the expression of HKGs between *T*. *parva* Muguga and 7014. The results from the first run are presented in panel A and results from the second run in panel B.

The Student’s *t*-test analysis for both inter- and intra-assay variation revealed that there was no significant variation in expression of the HKGs between *T*. *parva* Muguga and 7014, shown by the p values greater than 0.05 and the coefficient of variation percentage low (<2) for all the genes tested (Fig 4A and 4B). Inter-assay (run to run) variation analysis illustrated low coefficient of variation percentage in all five HKGs (Table 3).

**Table 3:** Inter-assay variations analysis as determined by Student’s *t*-test in expression of the five candidate reference genes for *T*. *parva* Muguga and 7014.

Gene	Run 1 mean Ct	Run 2 mean Ct	Standard deviation	Coefficient of variation (%)
**28S rRNA**	14.54018	14.68575	0.072788	0.49
**Cytochrome b**	15.76403	15.71166	0.026	0.17
**Beta-actin**	20.05952	19.75942	0.150047	0.75
**GAPDH**	23.63574	21.75894	0.9384	4.1
**F6P**	21.03138	20.59538	0.218001	1.1

### Expression analysis of genes differentially expressed in *T*. *parva* Muguga and 7014 isolates

**Fig 5:**
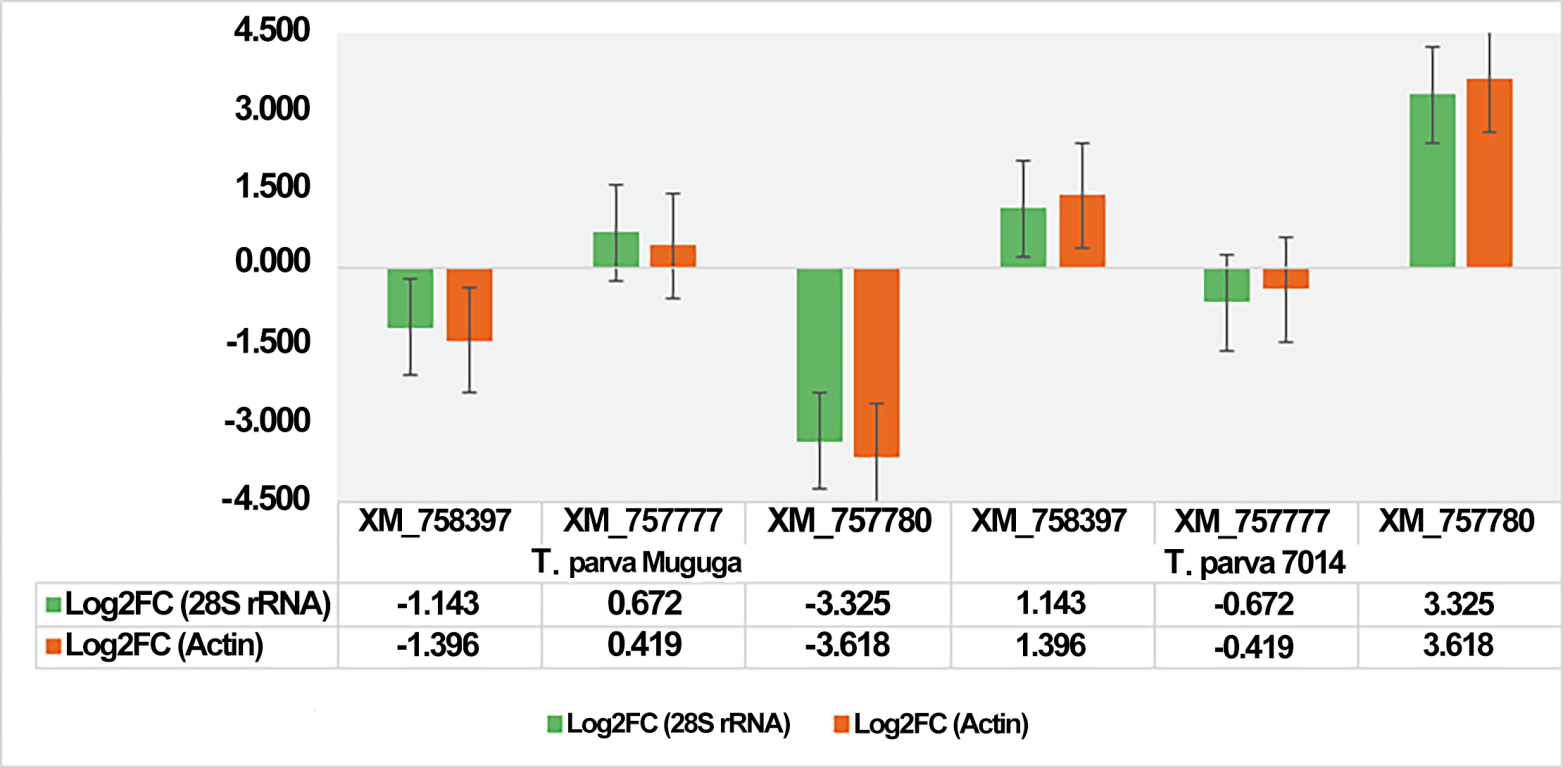
The comparison of expression profiles of the three differentially expressed genes based on the log2 fold change values obtained from qPCR analysis when the data was normalized using 28S rRNA or β-actin as endogenous gene controls.

The expression profiles of the three differentially expressed genes were analyzed using the comparative C_T_ method in which each gene was first normalized using β-actin and then 28S rRNA. The log2 fold change values obtained from the two datasets, i.e. the qPCR results normalized using β-actin and those normalized with 28S rRNA, showed that the results were comparable (Fig 5).

## Discussion

The *Theileria* genus belongs to the phylum Apicomplexa, together with other parasite genera, such as *Eimeria*, *Plasmodium*, *Sarcocystis* and *Toxoplasma*, responsible for significant diseases of animals and humans [29–32]. Reportedly, for regulation of gene activity, apicomplexan parasites rely on epigenetic mechanisms, as they are deficient in key transcription factors commonly found in eukaryotes [33]. Hence, the studies of gene regulation and expression in these parasites have drawn the interest of researchers. As mentioned earlier, qPCR has become the method of choice for quantification of mRNA transcription. However, analysis using this rapid and reliable technique can be compromised by error if an appropriate reference gene is not included in the assay thus affecting the resulting quantification analysis [15,16].

*Theileria parva* parasites occur in two groups, cattle-derived and buffalo-derived parasite isolates. Parasites of the former group cause ECF while those belonging to the latter are responsible for Corridor disease [22]. Hence, the expression of the selected HKGs was evaluated in isolates that represent both groups. The evaluated HKGs were detected in the schizont developmental stage of the parasite and the expression profiles of the two most stable HKGs were comparable in both *T*. *parva* stocks investigated. The expression of three of the evaluated HKGs was found to be unstable demonstrating that not all HKGs may be suitable reference genes. Normally, a suitable reference gene should have constant expression between samples investigated while it is expressed in detectable quantities in the target individual or tissue [5, 34, 35].

A number of parameters are fundamental to the success and adaptability of a qPCR assay for gene expression analysis [36]. Firstly, it is essential that the qPCR assay should have the capability to recognize multiple strains of the parasite with a high level of specificity. In addition, optimized performance with a single thermal cycling protocol and reaction conditions is necessary for utilizing the system as a qPCR array, to allow quantification of multiple gene targets on a single plate. Lastly, it is imperative for the qPCR system to quantify RNA levels accurately across a wide linear range of template concentrations with minimal intra- and inter-assay variability [36]. In accordance with the parameters stated above, the specificity of the *T*. *parva* HKG primers used for amplification of the gene target regions in this study was positively confirmed by specific melting peaks and amplicon sizes. The specific primers also successfully detected the target genes in both *T*. *parva* stocks. Intra- and inter-assay variation analysis of the investigated HKGs showed no significant variation in the expression of these genes between the two *T*. *parva* isolates, *T*. *parva* Muguga and *T*. *parva* 7014, with the p values being greater than 0.05 and the coefficient of variation percentage being low (<2) for all the genes tested. The absence of significant intra-or inter-assay variation enables plate-to-plate comparisons between results obtained and provides statistical significance when examining replicate datasets.

Quantitative studies are commonly undertaken to compare RNA expression in different experimental conditions. The quantification of specific RNA transcripts is carried out by comparing their expression against that of the HKG transcripts [5]. It is thought that there are minimal fluctuations in the synthesis of HKGs in comparison to that of other gene transcripts; hence, they are considered to be constant and reliable reference genes in many quantitative studies. However, numerous studies have shown that even HKGs can be variable under certain conditions [5]. In our study, the expression stability analysis by qPCR and RefFinder showed that, of the five *T*. *parva* HKGs (β**-**actin, GAPDH, 28S rRNA, cytochrome b and F6P) evaluated as candidate endogenous genes for gene expression studies of this parasite, only two, 28S rRNA and β**-**actin, had stable expression profiles, in the two parasite stocks investigated. The fact that three of the commonly used HKGs were shown to have variable expression levels between the two *T*. *parva* isolates studied, highlights the danger of using HKGs as endogenous reference controls without proper evaluation, which takes into consideration the cell type, tissue type and parasite stock (as in the case of our study); such an oversight can lead to erroneous results [37,38]. Our results further confirm that the expression of some HKGs may be variable under different conditions, as has been shown by other studies [39]. The actin and fructose bisphosphate aldolase (FBA) genes have been used to normalize expression profiles of genes encoding subtelomeric variable secreted proteins (*SVSP*s), investigated by qPCR in *T*. *parva-*infected cell lines generated from Marikebuni and Muguga parasite strains [40]. In *T*. *annulata*, the 18S rRNA gene has been used and later confirmed to be the most stable in expression compared to GAPDH, β-actin, PRKG1 (protein kinase cGMP-dependent, type I) and TATA box binding protein (TBP) [41,42]. This variability in the use of HKGs with different *Theileria* species and/or parasite strains highlights the necessity to perform a proper evaluation of HKGs for each experiment in case the parasite strains/species under investigation or experimental conditions may affect the expression stability of these genes. Generally, HKGs are involved in processes that maintain the proper functioning of the cell hence their expression is expected to be constant in different individuals, tissues or cells; however, they are also reported to have other functions [43–45]. The latter could explain why certain experiment or clinical conditions induce differential expression of HKGs [45]. Hence, some of these HKGs have been reported to be regulated occasionally [46].

Fundamentally, an endogenous control gene should be stably expressed while it is expressed at detectable levels, in the target tissue [47], which is typical of HKGs, over and above the fact that they are present in all cells, hence they make ideal endogenous control gene candidates [35,48]. The HKGs remain attractive for this role since, as a principle, the endogenous control gene expression levels should vary from that of the target gene when exposed to different experimental or clinical conditions; qPCR can quantify RNA over a wide dynamic range thus ensuring accurate measurement of target gene expression levels [49]. However, not all HKGs remain stably expressed under all experimental or clinical conditions; thus, each housekeeping gene should be evaluated for the suitability for use as an endogenous control for specific experimental or clinical conditions [35]. The use of two reference genes in combination is advised to be more accurate as oppose to using a single most stable gene [50]. It should also be noted that the criteria for stability ranking differ from program to program; hence, the outcome of the stability rankings varies according to the program used. It is for this reason that the RefFinder tool integrates the different programs and gives a recommended comprehensive ranking. Therefore, based on this comprehensive analysis between the two *T*. *parva* isolates investigated, 28S rRNA and β-actin were identified as suitable endogenous control genes for studies that involve gene expression analysis of *T*. *parva*.

## Conclusion

Either the host or the parasite HKG can be used to normalize data in qPCR analysis of the parasite gene expression in *Theileria*-infected cell lines because the infection in transformed cell lines is maintained by the simultaneous division of the infected cell and the parasite. However, this study was aimed at targeting the parasite-specific HKGs, so that the selected genes can be used to normalize the qPCR data in gene expression studies not only *in vitro*, but *in vivo* as well. Host HKGs may not be suitable in *in vivo* studies since not all the host cells are infected by the parasite.

All the HKGs evaluated in the current study have been widely used as endogenous controls, however, to our knowledge, this is the first report on their evaluation for expression stability in *T*. *parva*. Our results indicate that expression stability varies between the *T*. *parva* HKGs that were assessed. Of the five candidate endogenous control genes evaluated, only two, β**-**actin and 28S rRNA, showed the required expression stability for use in nucleic acid quantification studies for *T*. *parva*.
